# Glycemic control in critically ill infants and children: achieved quality of control in daily clinical practice in Leuven after a RCT

**DOI:** 10.1186/cc9823

**Published:** 2011-03-11

**Authors:** E Voets, T Van Herpe, L Desmet, D Vlasselaers, P Wouters, G Van den Berghe

**Affiliations:** 1 UZ Leuven, Belgium

## Introduction

A large RCT of our research group demonstrated that targeting age-adjusted normal fasting blood glucose concentrations with insulin infusion improves outcome in critically ill infants, children and adults [1-3]. Tight glycemic control according to the Leuven guideline has been implemented as a standard of care in all Leuven ICUs. This study aims to document the quality of glycemic control in daily clinical practice in the Leuven pediatric ICU (PICU).

## Methods

We performed a retrospective data analysis on all pediatric patients admitted to the Leuven PICU over a 12-month period, from 1 January 2009 to 31 December 2009.

## Results

One hundred and forty-two of the 333 PICU admissions (43%) were infants (<1 year) and 191 of 333 (57%) were children (1 to 16 years). We obtained a total of 12,208 blood samples in the infant group. The mean blood glucose level per infant was 98 mg/dl, the median was 86 mg/dl (interquartile range 67 to 111 mg/dl). Forty-six infants (32%) experienced at least one hypoglycemic period. Hypoglycemia (<40 mg/dl) was noted in 168 (1.4%) of the samples, and 37 samples (0.3%) were extreme hypoglycemic (≤30 mg/dl). A total of 8,008 blood samples were taken in the children's group. The mean blood glucose level per child was 116 mg/dl, the median was 103 mg/dl (interquartile range 88 to 125 mg/dl). Sixteen (8%) children experienced at least one hypoglycemic period. Twenty-two samples (0.3%) were hypoglycemic (<40 mg/dl) and three samples (0.04%) were extreme hypoglycemic (≤30 mg/dl).

## Conclusions

Even outside the setting of a RCT, the blood glucose control achieved in clinical practice mimicked that during the study on tight glycemic control in critically ill infants and children [3]. The risk of hypoglycemia was even lower than during the RCT. These outstanding results were achieved by standardized management by experienced nurses who were allowed to make anticipative decisions. The principles of managing tight glycemic control in the PICU will be shared onsite.
